# From microscale to microbial insights: validating high-throughput microvolume extraction (HiMEx) methods for marine microbial ecology

**DOI:** 10.1093/ismeco/ycaf218

**Published:** 2025-11-25

**Authors:** Marjan Ghotbi, Mitra Ghotbi, Elisa D’Agostino, Maarten Kanitz, David M Needham

**Affiliations:** Ocean EcoSystems Biology Unit, Marine Ecology Division, GEOMAR Helmholtz Centre for Ocean Research Kiel, Kiel, Schleswig-Holstein 24148, Germany; Faculty of Mathematics and Natural Sciences, Kiel University, Kiel, Schleswig-Holstein 24118, Germany; Department of Biology, Middle Tennessee State University, Murfreesboro, TN 37132, USA; Ocean EcoSystems Biology Unit, Marine Ecology Division, GEOMAR Helmholtz Centre for Ocean Research Kiel, Kiel, Schleswig-Holstein 24148, Germany; Faculty of Mathematics and Natural Sciences, Kiel University, Kiel, Schleswig-Holstein 24118, Germany; Ocean EcoSystems Biology Unit, Marine Ecology Division, GEOMAR Helmholtz Centre for Ocean Research Kiel, Kiel, Schleswig-Holstein 24148, Germany; Ocean EcoSystems Biology Unit, Marine Ecology Division, GEOMAR Helmholtz Centre for Ocean Research Kiel, Kiel, Schleswig-Holstein 24148, Germany; Faculty of Mathematics and Natural Sciences, Kiel University, Kiel, Schleswig-Holstein 24118, Germany

**Keywords:** metagenomics, viruses, rRNA gene sequencing, marine, microvolume extraction

## Abstract

Extracting and directly amplifying DNA from small-volume, low-biomass samples would enable rapid, ultra-high-throughput analyses, facilitating the study of microbial communities where large-volume sample collection is challenging. This can aid where ‘conventional’ filtrater-based methods miss capturing smaller microbes, or where microscale variability matters, such as the ocean. Here, we develop and validate physical and chemical-based DNA extractions from microvolumes with universal rRNA gene amplicons and metagenomic sequencing of all domains and viruses, on natural surface seawater and experimentally manipulated marine waters. Compared to 500-mL filter-based extraction, direct PCR of 3 μL of lysate from seawater microvolume extractions ranging from 100–1000 μL consistently captured comparable microbial community composition and diversity, with reliable amplification and little to no contamination. Metagenomic results of 10 μL lysates from 15 microvolume samples (100 μL) captured 83 high- and draft-quality, diverse bacterial genomes and 430 complete, high and medium quality viral contigs. Our approach enables scaling of rRNA gene sequencing and metagenomic library prep for high-throughput experimentation for a fraction of the cost of conventional methods and builds upon existing microvolume approaches by removing unnecessary expenses, like excess plasticware and expensive bead clean-up. The method expands opportunities for more comprehensive microbial community monitoring and controlled laboratory experiments by facilitating higher sample numbers and lowering sample volume needs. However, its potential bias against Gram-positive bacteria should be considered when applying to environments where these taxa are abundant.

Molecular characterizations, both taxonomic and genomic, of microbial communities is essential for understanding microbial diversity and dynamics. Traditionally, this involves collection of substantial amounts of biomass that require significant time, effort, and resources to collect, extract, and analyse. However, if sample biomass could be scaled down to be performed in microvolumes, then sample numbers could be scaled up tremendously. Applications of such methods can include high-throughput experimentation that enables testing of numerous ecological factors within a single experiment (with high replication and greater statistical power, while also reducing costs), situations where sample volume or biomass has limitations and studies where microscale diversity is important, such as the phycosphere of microalgae [1]. Although innovative microvolume extraction methods [2, 3] have achieved success in the molecular characterization of community composition, a bottleneck, specifically for high-throughput studies (e.g. 1000s of samples), remains the relatively high cost of DNA purification. Direct amplification of extracted DNA or a minimal, yet cost-effective purification step would be a preferred alternative if high-throughput analyses are required since it excludes the expensive bead-purification or centrifugation. Here, we develop and evaluate different microvolume extraction methods, coupled with both amplicon and metagenomic analysis. We demonstrate that this approach facilitates high-throughput lab experiments and captures diversity across all domains and viruses, to provide insight into microbial dynamics and interactions.

For High-throughput Microvolume Extraction (HiMEx) methods, we used either physical extraction through thermal shock (i.e. freeze–thaw, FT) and/or a combination of physical and chemical extraction which included the addition of the lytic enzyme alone to the physical disruption (i.e. FT + proteinaseK, FTP), or a combination of lytic enzyme and a surfactant (i.e. FTP + IGEPAL, FTPIG) [3] (Fig. 1A). For testing the efficacy of the HiMEx methods, we first compared the methods via amplicon analysis from natural surface seawater samples (Baltic Sea) collected bimonthly at seven timepoints during August to November 2022 (54°19.813′ N, 10°8.993′ E). Across these samples, total eukaryotic phytoplankton, prokaryote, and virus-like-particle abundance ranged from 2.92–179.63 × 10^2^, 0.97–7.56 × 10^6^, and 0.56–5.04 × 10^7^ per mL, respectively (Table S1). Quadruplicate samples of 500 mL filtered seawater (0.2 μm, *n* = 27) for ‘conventional’ extraction and microvolume whole seawater samples (100 (n = 30), 200 (*n* = 6), 400 (*n* = 6), 1000 (*n* = 18) μL) for HiMEx methods were used for comparison. An incomplete factorial design across the seven timepoints was applied, where a subset of HiMEx methods (FT, FTP, FTPIG) and volumes (100, 200, 400, 1000 μL) were tested in different months (Fig. S1) and compared with the conventional method (control). Treatments were analysed in triplicate or quadruplicate (with the exact number of replicates, extraction methods, volumes, and exceptions indicated in Table S2). The range of microvolumes were used to test whether the volume of the extracted sample can affect community structure, due to concerns that microscale spatial variability may add variability to smaller samples [1]. All samples were immediately stored at −80°C. For filter-based ‘conventional’ extraction, samples were extracted via DNeasy Plant Kit (QIAGEN) with some modifications (Supplementary Information). HiMEx methods (three μL of lysate) and ‘conventional’ (1 ng of DNA) extractions were analysed through amplicon sequencing using universal primer set that amplifies prokaryotic 16S, chloroplast 16S, and 18S rRNA genes simultaneously [4]. We also analysed mock communities with 10**×** fewer cells than our lowest seawater sample (Fig. S2, Table S3).

**Figure 1:**
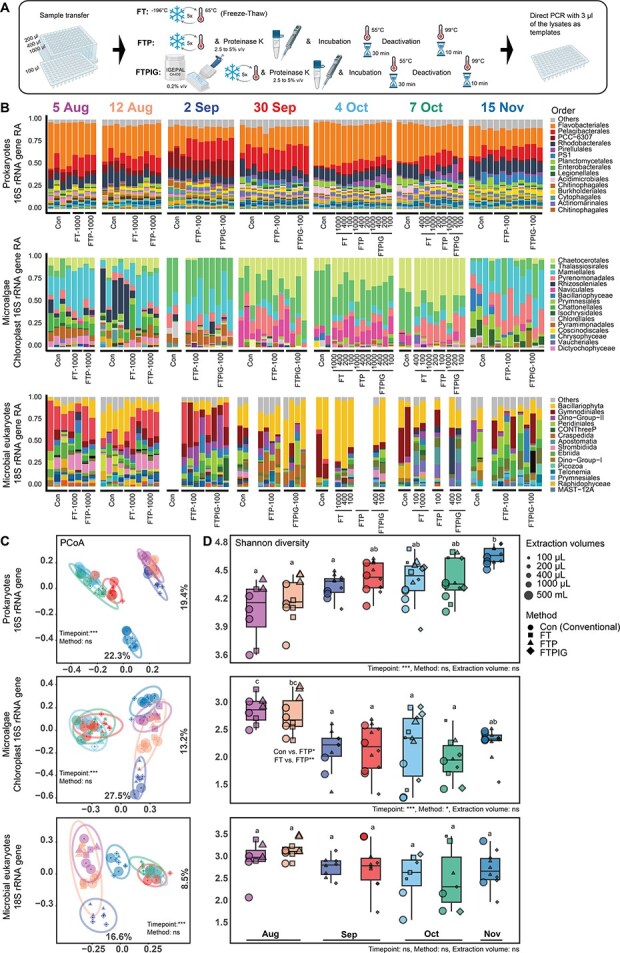
(A) HiMEx DNA extraction methods rely on either physical extraction through thermal shock (FT) or a combination of physical and chemical extraction which includes addition of the lytic enzymes alone to the physical disruption (FTP), or a combination of lytic enzyme and a surfactant (FTPIG). After extraction 3 μL of each lysed sea water sample was used as a template for amplification via direct PCR. Community composition via HiMEx and ‘conventional’ 500-mL filter-based DNA extraction are highly concordant. (B) Microbial community composition for prokaryotes, chloroplasts, and microbial eukaryotes consolidated at the order level, top 30 orders are coloured in the barplots and the rest combined into ‘other’. Top 15 orders are labelled next to their colour codes (complete colour code presented in Fig. S3). The absence of a bar in each timepoint indicates that sequence reads did not meet the rarefaction threshold (1000 read depth for 16S and 100 for chloroplast and 18S rRNA genes were considered for all visualization purposes). We note, the rarefaction threshold of 100 for chloroplast 16S and 18S was not typically saturated but was utilized to allow inclusion of a higher number of samples (Fig. S4). (C) PCoA of prokaryotes, chloroplast, and microbial eukaryotes is shown where colours represent different timepoints, shapes indicate the extraction methods, and the size of the points reflects the extraction volumes. The ellipses represent 95% confidence intervals around the clusters, and the results of PERMANOVA are indicated (Tab S5.  A-C). To examine the impact of the rarefaction depth to the chloroplast 16S analysis, we repeated the PERMANOVA tests at a depth of 500 reads which yielded similar results to the 100 read rarefaction (Tab S5-B1). However, for the 18S dataset it was not feasible due to removal of conventional samples from three timepoints. (D) Shannon diversity across conventional and HiMEx showing an increasing trend in prokaryotes over time from summer to winter vs a decreasing trend in microbial eukaryotes, perhaps detecting a seasonal transition. Linear mixed effect models [21] were used to assess the effect of different extraction methods and volumes on Shannon diversity while accounting for the fixed effect of sampling timepoints (mainly driven by temperature, monthly periods and salinity, Table S5. A1–C1), which showed similar and comparable results between HiMEx and conventional extraction (Table S8. A–F). Letters on top of each box plot represent statistical groupings based on pairwise comparisons. Timepoints with the same letter are not significantly different. The only case of significant difference between the extraction methods (12 August) is indicated.

87% of lysates of HiMEx methods were successfully amplified (Table S2), while blanks showed no amplification. In addition, all mock community samples were also successfully amplified (Table S2). Although intensity of amplification varied, after normalization and pooling, sequence reads were similar between conventional and HiMEx methods (Table S4). Between the methods, similar taxonomic diversity and relative abundances were observed across prokaryotic, microalgae and microbial eukaryotes (Fig. 1B), with community similarity clustering Principal Coordinate Analysis (PCoA) over time being observed across all domains (Fig. 1C). To further examine the statistical similarity between community composition via conventional and HiMEx methods we used PERMANOVA. Since we used an incomplete factorial design, balanced subsets of the data were established for evaluation (Fig. S1, Table  S2). Across all subsets, we found that DNA extraction methods and volumes were statistically similar, and samples were mainly clustered according to seasonal changes in temperature and salinity (Table S5.  A–C). To examine for subtle differences between extraction methods within each timepoint, we also performed pairwise comparisons and did not detect differences between conventional and HiMEx (Table S5–D1), though this analysis has less power than the more comprehensive analysis. Replicate variability was observed in both prokaryotes and microbial eukaryotes but was higher in the latter (Table S6). This might be due to comparatively increased stochasticity of eukaryotic cells and their attached bacteria [5] within microvolumes resulting from their lower overall cell abundances (Table S1). Potential seasonal stochasticity across timepoints together with the use of universal primers (Table S7) likely contributed to the lower recovery of eukaryotic sequences (Table S2). Thus, if eukaryotes are of special interest, 18S-specific primers [6, 7] may yield better results. In terms of alpha diversity, across all conventional and HiMEx samples the main driver was time and there was a significant impact observed between extraction methods when considering all data together, (Fig. 1D, Table S8.  A–F). However, pairwise comparison, indicated no significant difference (except for 12 August in chloroplast data, Fig. 1D), thus we could not pin-point differences potentially because the impact is subtle and a higher number of replicates may be needed. Altogether, all HiMEx methods showed comparable results with conventional extraction. However, the FTPIG method produced the most consistent results regarding replicates variability (Table S6). Among the tested volumes, 100 μL samples demonstrated high success rates and reproducibility, while also being the most practical in terms of handling time (for instance extraction, including incubation, can be carried out in a PCR plate), storage space, cost, and plastic waste. Hence, the combination of FTPIG with 100 μL samples is our most recommended method. For this HiMEx method (FTPIG-100 μL) the main compositional differences to the conventional extraction were driven mainly by ASVs of higher relative abundances from Pelagibacterales, Pirellulales and Legionellales via the FTPIG-100 μL method versus Pseudomonadales, Flavobacteriales, Rhodobacterales, Planctomycetales, Chthoniobacterales, and Chitinophagales via the conventional method (Supplementary Information, Table S9.  A–C). Despite the strong clustering between conventional and FTPIG-100 μL, these differences could reflect either variation in extraction efficiency, or differences between whole seawater and 0.2 μm filtered fraction of seawater. For instance, *Pelagibacter* as an ultramicrobacterium can potentially pass through 0.2-μm-pore-size filters [8–10].

After establishing that recovery of community structure is highly similar across cellular communities between conventional and HiMEx approaches, we sought to also establish its utility for shotgun metagenomics through a microcosm phytoplankton-enrichment experiment carried out over seven days (Fig. 2A). We utilized the conventional extraction for the Day 0 metagenome and HiMEx-FTPIG-100 μL for experimental timepoints of Days 1, 2, 3, and 7. Via a bead-based transposome library preparation approach (Hackflex) [11], conventional extraction metagenomes were produced from 1 ng of DNA, whereas HiMEx-FTPIG-100 μL extraction metagenomes were produced from 10 μL of AMPure bead-purified lysates. The bead-purification was not strictly required for amplification (Fig. S5), but nevertheless served to increase input and fragment size for the metagenomic sequencing (at marginally increased expense, Table S10-A). All samples from both conventional extraction and FTPIG-100 μL yielded similarly high-quality assemblies in terms of overall assembly metrics (Fig. 2B), MAG-quality and quantity (Fig. 2C). Each method recovered diverse MAGs (Fig. 2D, Fig. S6). Conventional extraction obtained 55 MAGs (range 23–32, n = 2 x 500 mL) with 33 representatives, while HiMEx yielded 83 (range 1–18, n = 15 x 100 μL) with 35 representatives. MAG completeness differed slightly across conventional extraction and FTPIG-100 μL (Kruskal–Wallis H(4) = 10.5, p = 0.033) (Fig. S7, Table  S12-A), but pairwise comparisons revealed no statistically significant differences. The number of contigs per MAG did not significantly differ among extraction methods (Kruskal–Wallis H(4) = 7.57, p = 0.109) (Fig. S7, Table  S12-B). Meanwhile, viral genome (contig) quality and quantity was higher from FTPIG-100 μL relative to the conventional method, owing most likely to removal of free viruses from the conventional filter-based approach (Fig. 2E, Table S11-B, Table  S14).

**Figure 2:**
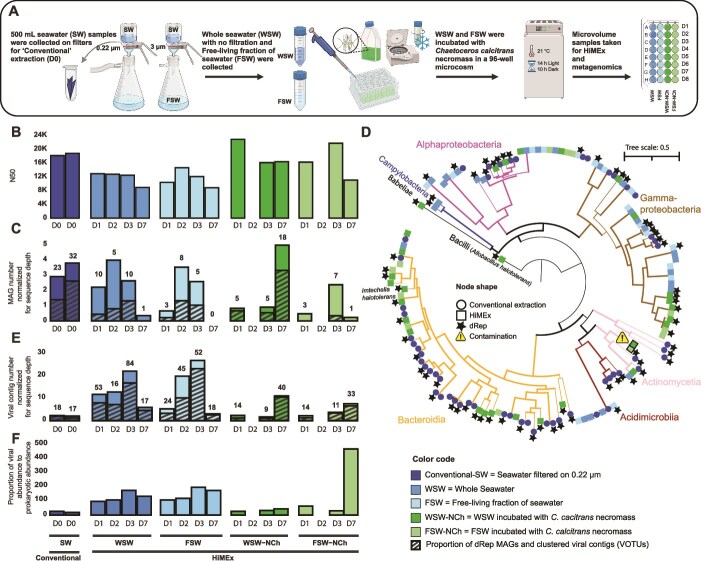
(A) a pilot high-throughput microcosm experiment investigated the impact of microalgal necromass (bloom demise) on multi-domain marine microbial community composition and diversity. Whole seawater and the free-living fraction (<3 μm) of surface Baltic Sea were incubated with *Chaetoceros calcitrans* necromass (obtained after gentle centrifugation and freeze–thaw of cells). Incubation was done in a 96-well microcosm at 21°C, 14 hours light and 10 hours dark for eight days. 100-μL daily samples were collected for metagenomic analysis. D0, D1, D3, D7 refer to sampling timepoints of the experimental sampling (e.g. Day 0, Day 1). (B) Overall assembly quality from HiMEx was comparable to that of the conventional extraction based on the total assembly N50. (C) Recovery of high- and draft-quality MAGs via conventional and HiMEx approaches were comparable after normalizing for sequence depth. In order to account for sequencing depth in MAG recovery, their total numbers were divided by total sequencing depth. In addition, the total numbers of MAGs are also presented on top of each bar (Table S11-A). Across all samples, 138 high and draft quality MAGs [22] were produced (55 and 83 from ‘conventional‘ and HiMEx, respectively), with 68 remaining after dereplication (indicated by black hatch patterns) (33 and 35 from ‘conventional’ and HiMEx, respectively) (Table S11-A, Table  S12-C). (D) Phylogenomic tree of all MAGs constructed from conventional and HiMEx approach was based on 25 ‘Bacteria_and_Archaea’ single copy gene HMM set protein sequences. Representative MAGs remaining after dereplication whereby MAGs are selected for higher quality and lower redundancy are highlighted with stars (dRep star indicates the highest quality MAG among essentially identical genomes on the same branch). Blanks for HiMEx did not produce any contigs >5 kb, so MAGs were examined for contaminants list from 16S (Table S13) which identified two *Cutibacterium* as potential contaminants, which were excluded from further analyses. Two samples of WSW-NCH and FSW-NCH from timepoint D2 that showed contamination were also removed from further analysis. In both methods we spiked two bacterial species (*Imtechella halotolerans* and *Allobacillus halotolerans*) within a range of an estimated 0.1 to 10% of the cell count to the sample before extraction to help with absolute quantification [23] (supplementary information), however, we only could retrieve high quality MAGs of these spiked bacteria from HiMEx metagenome amplification. (E) Excluding proviruses, 48 putatively complete circular viral genomes (1 vs 47) and 417 high- and medium- quality viral contigs were recovered from conventional and HiMEx samples (34 vs 383), with 344 VOTUs (26 vs 318) remaining after clustering (black hatches) (Table S11-B, Table  S14). Similar to bacterial MAGs, to account for sequencing depth, the total number of viral contigs was divided by total sequencing depth (Table S11-B). The total number of viral contigs are also presented on top of each bar (Table S11-B). Higher ratios of coverage-based viral to bacterial abundance were detected in whole seawater and free-living fraction of seawater compared to the conventional extraction (Table S15). Together, these results demonstrate valuable recovery of viruses from HiMEx.

Our findings from amplicon analysis indicate that HiMEx methods produce results for prokaryotes, microalgae, and microbial eukaryotes comparable to a ‘conventional’ filter-based method. Additionally, it offers an innovative, efficient, and cost-effective approach for capturing viral diversity without requiring large sample volumes or the filtration and concentration steps [12]. This method captures small microbes and extracellular DNA that are often missed by filter-based methods. Extracellular DNA is comprised of three main components: protein-encapsidated viruses, extracellular vesicles, and free DNA [13–16], however, their sampling via conventional concentration methods is perhaps thought to require large sample volumes, additional filtration, cost and effort [14–17]. HiMEx methods can capture these extracellular DNA as part of whole seawater. However, at present HiMEx methods cannot separate them from cellular fraction or distinguish between DNA originating from free DNA vs protein-encapsidated viruses, and extracellular vesicles. Hence, a potential consideration is that results from HiMEx methods may include sequences from free-DNA relative to filtration-based methods, along with the increased detection of viruses we observed. Since the proportion of vesicles and free DNA tends to increase with depth [15], this limitation may pose a greater concern in certain environments, such as water samples from deeper depths [18]. Free DNA may be addressed by additionally treating the sample with DNase prior to the physical lysis step [19], though this treatment needs further investigation. We were not able to directly compare to viromics datasets or to other methods targeting extracellular DNA. Thus, while we did demonstrate high recovery of viral genomes from 10 μL of lysate via HiMEx, potential biases or differences, due to e.g. filter-based size selection and sample volume, remains to be characterized.

In summary, HiMEx methods offer an approach for advancing high-throughput, microscale taxonomic and genomic studies in microbial ecology. Though all HiMEx methods performed quite well overall, we recommend the HiMEx method, FTPIG-100 μL, based on a combination of its performance and scalability. Like the other HiMEx methods, the main advantage for this method is that it (i) decreases costs compared to previously published methods for amplicon analysis and high-throughput experimentation (Table S10-A), (ii) allows the extraction of small sample volumes compared to conventional approaches, (iii) obtains sequences from all domains and viruses without the need for arduous fractionation, and (iv) saves a large amount of time (Table S10-B). To the latter point, manually extracting 96 samples in a plate-format with the recommended method takes around 15 minutes hands-on time (+40 minutes of incubation). The workflow could be adapted to robotics if ultra-high-throughput is needed, though the physical fractionation by freezing (via liquid-nitrogen or similar cooling system) may require special modules or considerations. Beyond these advantages, potential further considerations are that (i) analyses may be sensitive to long-term storage and extracts may be less robust due to the potential that lysates degrade faster than purified DNA (not yet investigated), (ii) unpurified lysates may contain inhibitors depending on the environment (such as humic substances [20]), which potentially may be minimized through template volume trade-offs, (iii) for communities with lower biomass than those of the surface seawater and the mock communities tested (10^4^), amplification and contamination may become an issue, (iv) where eukaryotic taxonomic diversity is of priority, the use of eukaryote-specific primers could be beneficial, (v) while we focused on short-reads and Illumina sequencing the input amounts as well as high number of freeze–thaw (which potentially could be reduced) may challenge the ability to do long-read sequencing via HiMEx, and (vi) while we validated HiMEx for marine microbial ecology its potential bias against some Gram-positive bacteria should be considered when applying to environments where these taxa are abundant. Future work applying HiMEx across a wider range of environmental samples and experimental conditions will help further validate its robustness and adaptability, providing a stronger foundation for its broader application. HiMEx offers the ability to scale microbial community composition and genomics study in terms of throughput, and should facilitate biological questions related to microscale variability, high-throughput experimentation, and access to molecular data where sample volume is limited.

## Supplementary Material

Ghotbi_etal_Supplementary_Information_ycaf218

Ghotbi_etal_SupplementaryTables_ycaf218

## Data Availability

Raw reads are available from SRA under BioProject IDs PRJNA1365321 and PRJNA1365741. Scripts for analysis and figures are available via https://github.com/Marjan-Ghotbi/HiMEx. MAGs and viral contigs (including VOTUs) are available via FigShare DOI: https://doi.org/10.6084/m9.figshare.30328354.

## References

[1] Seymour JR, Amin SA, Raina J-B. et al. Zooming in on the phycosphere: the ecological interface for phytoplankton-bacteria relationships. *Nat Microbiol* 2017;2:17065. 28555622 10.1038/nmicrobiol.2017.65

[2] Bramucci AR, Focardi A, Rinke C. et al. Microvolume DNA extraction methods for microscale amplicon and metagenomic studies. *ISME Commun* 2021;1:79. 10.1038/s43705-021-00079-z 37938281 PMC9723667

[3] Song F, Kuehl JV, Chandran A. et al. A simple, cost-effective, and automation-friendly direct PCR approach for bacterial community analysis. *mSystems* 2021;6:e0022421. 10.1128/msystems.00224-21 34581599 PMC8547444

[4] Yeh Y-C, McNichol J, Needham DM. et al. Comprehensive single-PCR 16S and 18S rRNA community analysis validated with mock communities, and estimation of sequencing bias against 18S. *Environ Microbiol* 2021;23:3240–50. 10.1111/1462-2920.15553 33938123

[5] Pernthaler J, Krempaska N, le Moigne A. Small-scale spatial beta diversity of bacteria in the mixed upper layer of a lake. *Environ Microbiol* 2023;25:1847–59. 10.1111/1462-2920.16399 37173811

[6] Balzano S, Abs E. Protist diversity along a salinity gradient in a coastal lagoon. *Aquat Microb Ecol* 2015;74:263–77. 10.3354/ame01740

[7] Amaral-Zettler LA, McCliment EA, Ducklow HW. et al. A method for studying protistan diversity using massively parallel sequencing of V9 hypervariable regions of small-subunit ribosomal RNA genes. *PLoS One* 2009;4:e6372. 10.1371/annotation/50c43133-0df5-4b8b-8975-8cc37d4f2f26 19633714 PMC2711349

[8] Nakai R . Size matters: ultra-small and filterable microorganisms in the environment. *Microbes Environ* 2020;35:ME20025. 10.1264/jsme2.ME20025 PMC7308576 32493880

[9] Yang Y, Nagata T. Viral production in seawater filtered through 0.2-μm pore-size filters: a hidden biogeochemical cycle in a neglected realm. *Front Microbiol* 2021;12:774849. 10.3389/fmicb.2021.774849 34867916 PMC8637275

[10] Lanclos VC, Rasmussen AN, Kojima CY. et al. Ecophysiology and genomics of the brackish water adapted SAR11 subclade IIIa. *ISME J* 2023;17:620–9. 10.1038/s41396-023-01376-2 36739346 PMC10030771

[11] Gaio D, Anantanawat K., To J et al. Hackflex: low-cost, high-throughput, Illumina Nextera flex library construction. *Microb Genom* 2022;8:8. 10.1099/mgen.0.000744 PMC8914357 35014949

[12] Hurwitz BL, Deng L, Poulos BT. et al. Evaluation of methods to concentrate and purify ocean virus communities through comparative, replicated metagenomics. *Environ Microbiol* 2013;15:1428–40. 10.1111/j.1462-2920.2012.02836.x 22845467 PMC3655615

[13] Biller SJ, McDaniel LD, Breitbart M. et al. Membrane vesicles in sea water: heterogeneous DNA content and implications for viral abundance estimates. *ISME J* 2017;11:394–404. 10.1038/ismej.2016.134 27824343 PMC5270575

[14] Schatz D, Schleyer G, Saltvedt MR. et al. Ecological significance of extracellular vesicles in modulating host-virus interactions during algal blooms. *ISME J* 2021;15:3714–21. 10.1038/s41396-021-01018-5 34083751 PMC8630046

[15] Linney MD, Eppley JM, Romano AE. et al. Microbial sources of exocellular DNA in the ocean. *Appl Environ Microbiol* 2022;88:e0209321. 10.1128/aem.02093-21 35311515 PMC9004351

[16] Ahmed AAQ, McKay TJM. Environmental and ecological importance of bacterial extracellular vesicles (BEVs). *Sci Total Environ* 2024;907:168098. 37884154 10.1016/j.scitotenv.2023.168098

[17] Sun G, Xiao J, Wang H. et al. Efficient purification and concentration of viruses from a large body of high turbidity seawater. *MethodsX* 2014;1:197–206. 10.1016/j.mex.2014.09.001 26150953 PMC4473021

[18] Corinaldesi C, Tangherlini M, Manea E. et al. Extracellular DNA as a genetic recorder of microbial diversity in benthic deep-sea ecosystems. *Sci Rep* 2018;8:1839. 10.1038/s41598-018-20302-7 PMC5789842 29382896

[19] Villarreal JV, Jungfer C, Obst U. et al. DNase I and proteinase K eliminate DNA from injured or dead bacteria but not from living bacteria in microbial reference systems and natural drinking water biofilms for subsequent molecular biology analyses. *J Microbiol Methods* 2013;94:161–9. 10.1016/j.mimet.2013.06.009 23811209

[20] Sidstedt M, Rådström P, Hedman J. PCR inhibition in qPCR, dPCR and MPS-mechanisms and solutions. *Anal Bioanal Chem* 2020;412:2009–23. 10.1007/s00216-020-02490-2 32052066 PMC7072044

[21] Ghotbi M, Taghizadeh-Mehrjardi R, Knief C. et al. The patchiness of soil 13C versus the uniformity of 15N distribution with geomorphic position provides evidence of erosion and accelerated organic matter turnover. *Agric Ecosyst Environ* 2023;356:108616. 10.1016/j.agee.2023.108616

[22] Sieber CMK, Probst AJ, Sharrar A. et al. Recovery of genomes from metagenomes via a dereplication, aggregation and scoring strategy. *Nat Microbiol* 2018;3:836–43. 10.1038/s41564-018-0171-1 29807988 PMC6786971

[23] Ghotbi M, Stajich JE, Dallas JW. et al. Absolute abundance unveils Basidiobolus as a cross-domain bridge indirectly bolstering gut microbiome homeostasis. *ISME J* 2025;19:wraf150. 10.1093/ismejo/wraf150 40689579 PMC12400927

