# Supplementary material for: From microscale to microbial insights: validating high-throughput microvolume extraction (HiMEx) methods for marine microbial ecology
Source: ISME Commun. 2025 Nov 25;5(1):ycaf218. doi: 10.1093/ismeco/ycaf218 (PMC12721387; doi:10.1093/ismeco/ycaf218)
Supplement: Ghotbi_etal_Supplementary_Information_ycaf218 [file ghotbi_etal_supplementary_information_ycaf218.pdf]

Supplementary Information for

From Microscale to Microbial Insights: Validating High-Throughput Microvolume Extraction Methods (HiMEx) for Microbial Ecology

Materials and Methods

Sampling for amplicon analysis

Seawater was collected via a bucket from the top meter of Kiel Fjord (54°19.813' N, 10°8.993' E) into a 4 L bottle. Immediately prior to any step of sample processing, the sample bottle was mixed to ensure homogeneity. For HiMEx, a subsample of 50 mL was taken in a centrifuge tube to enable gentle swirling of the water sample before pipetting of each microvolume sample. For the conventional method 500 mL was collected on membrane filters (Pall Supor, Fisher Scientific, 0.2 µm, 47 mm; Germany) and for microvolumes, 100 µL samples were individually pipetted into strips and 1 mL-samples into 2 mL microcentrifuge tubes, and all stored at -80°C until extraction. For high-throughput extraction, 100 µL HiMEx samples were transferred to PCR plates and 1 mL samples were subsampled and transferred in deep-well plates (200, 400, or 1000 µL). However, to facilitate high-throughput analysis, it is recommended to perform sample storage and extraction in the same plate, for further minimizing sample loss and reducing plastic use.

Conventional DNA extraction

Samples consisted of four replicate filters (except for timepoint 92 with three replicates; Fig. S1), and three blank replicates including DNA extraction reagents and filters were considered to identify contaminants. For 'conventional', filter samples, *Imtechella halotolerans* and *Allobacillus*

halotolerans (ZymoBIOMICS Spike-in Control I (High Microbial Load)) were added to each sample at roughly 5% (1 to 10%) of the cell count in the sample before extraction (specifically, assuming 1,000,000 cells per mL, 0.25 µL was used for spike-in). A modified extraction was performed based on the DNeasy Plant Kit (Qiagen). The DNeasy-based extraction method we used adds multiple freeze-thaws and a bead-beating step in addition to the DNeasy Plant Kit protocol in an effort to exhaustively extract DNA from all marine microbes. This physical lysis approach in addition to the usage of DNeasy Plant kit is based on [1] and [2] with our adaptations and full protocol as follows. First, filters were crushed, and then 50 µL of 0.1 and 0.5 mm Carl Roth beads and 750 µL of Lysis Buffer AP1 was added, followed by three rounds of freeze and thaw cycles in liquid nitrogen and 65°C water bath, and bead beating in Mixer Mill from Retsch at 30 Hz. Then, lysate was transferred to a 96-well plate, and 45 µL of Proteinase K was added followed by incubation for 30 minutes at 55°C. Then, 4 µL of RNaseA was added and incubated for an additional 10 minutes at 65°C. 130 µL of Buffer P4 was then added and incubated on ice for 10 minutes, followed by centrifugation to pellet debris. 650 µL of supernatant was added to another 96 well plate and 975 µL of Buffer AW1 added, followed by transfer to a NAB (AcroPrep Advance 96 Filter Plate Nucleic Acid Binding 1 mL well – Pall Corporation). DNA was collected on the column by placing the plate on a vacuum manifold and filtered at less than 250 mm Hg, until all the volume has been collected. Columns were washed 2x with 600 µL of Buffer AW2, and DNA eluted with 3x with 50 µL of pre-heated TE.

HiMEx extraction

Samples consisted of three or four replicates (Fig. S1). Spike-in cells were not added to environmental samples for amplicon analysis to ensure the method works with cells and

concentrations naturally occurring in marine water and to avoid misleading amplification due to addition of spike-in. All tubes, filter tips used during this protocol were sterile and DNA, RNase-Free. Molecular grade water and all reagents used except for the Proteinase K and DNA polymerase were crosslinked in a UVP crosslinker (CL-3000, Analytik Jena) on the highest energy setting for 1 hr prior to use [3]. The HiMEx was performed in a clean bench. Prior to sample processing, the workspace and clean bench were wiped with bleach (10%) and ETOH (70%). Samples were thawed on ice and vortexed.

Physical Lysis

Samples were heat-shocked for five cycles of freeze-thaw (FT), via rapid alternation between liquid nitrogen (-196 °C) and a 65 °C water bath (Fig. 1-A). Care was taken not to completely submerge the plate into liquid nitrogen nor the water bath to avoid contamination or sample loss. Samples were briefly vortexed to ensure thorough mixing. Either no centrifugation, or a brief, low speed spin was used to collect the volume at the bottom of the tube, while avoiding pelleting the cells and/or debris. Depending on the sample volume a thermoblock or thermocycler was used to heat samples at 99 degrees for 10 minutes. Finally, samples were briefly vortexed to mix thoroughly.

Chemical Lysis

FTP treatment (freeze-thaw, plus proteinase K)

Samples were similarly heat shocked for five cycles of freeze-thaw. Then, 5% v/v of Proteinase K (Qiagen, 19133) was added to each sample except for 1000-µL samples that received 2.5%, due to the cost considerations associated with high-throughput extraction at larger microvolumes.

Samples were incubated at 55 °C for 20 minutes. Then samples were briefly vortexed to mix and centrifuged very briefly at low speed to collect the reagents. Depending on the sample volume a thermoblock or thermocycler was used to heat samples to 99 °C for 10 minutes to deactivate Proteinase K. Finally, samples were briefly vortexed to mix (Fig. 1-A).

FTPIG treatment (freeze-thaw, plus proteinase K and IGEPAL)

Prior to the physical lysis step, IGEPAL 20% was prepared fresh by adding PBS (Phosphate-buffered saline) to IGEPAL® CA-630 (Sigma-Aldrich, I3021). Then 1% v/v of IGEPAL 20% was added to each sample (for instance 1 μL of IGEPAL 20% was added to the 100 μL sample). Samples were heat shocked for five cycles of freeze-thaw, and were briefly vortexed to ensure mixing. 5% v/v of Proteinase K was added to each sample except for 1000-μL samples that received 2.5%. Samples were incubated at 55 °C for 20 minutes. Then they were briefly vortexed to mix and collect the reagents. Samples were heated to 99 °C for 10 minutes to deactivate Proteinase K. Finally, samples were briefly vortexed to mix (Fig. 1-A).

rRNA gene PCR amplifications and sequencing

For rRNA gene amplicon analysis on 'conventional' samples, 1 ng of DNA extract was PCR amplified via a single PCR. In addition to the template specific primers (515F-Y GTGYCAGCMGCCGCGGTAA, 926R CCGYCAATTYMTTTRAGTTT [4]), the primers included TruSeq I5/I8 TruSeq sequencing adapters, eight bp indexes, and seven bp heterogeneity spacers (i.e., all "N") (Tab. S7). KAPA HiFi HotStart PCR Kit (Roche, KK2102) was used following manufacturer protocols. These primers were chosen because they are 'universal' for Bacteria, Archaea, and Eukaryotes (plus chloroplasts), and have been demonstrated via mock to

be among the best performing sets for each domain, even considering domain specific, within a single assay [5, 6]. PCR thermocycling began with an initial denaturation at 95 °C for five minutes, followed by 30 cycles of 20 seconds of denaturation at 98 °C, 15 seconds of annealing at 55 °C, and 1 minute of extension at 72 °C. A final extension step was performed at 72 °C for five minutes. The resulting PCR products were visualized via Agarose gel electrophoresis for a band size of ~525 bp for 16S and/or ~720 bp for 18S (in this case, bands were typically 16S). The number of successful amplifications for samples is indicated in Tab. S2; all blanks showed no amplification. Subsequently, PCR products and blanks were purified and size-selected using 0.8× (vol:vol) Agencourt AMPure XP magnetic beads (Beckman Coulter) and then pooled at approximately equimolar concentrations after quantification by Quant-iT PicoGreen dsDNA Assay Kit. The pooled library underwent an additional purification step using 0.8× (vol:vol) AMPure beads before sequencing. The blank samples, despite no amplification, were incorporated into the final sequencing libraries.

For all three HiMEx methods, PCR was carried out similarly as above except 3 µL of the lysate was used directly as a template for amplification without any purification steps. Additionally, 5% DMSO (Phusion® Hot Start Flex, New England Biolabs) was added to each reaction, and the PCR was cycled 38 times. Blank samples for different steps of extraction (mostly in triplicates) were included in amplification. All HiMEx PCR products were purified and size-selected using 0.8× (vol:vol) AMPure beads. and, along with PCR blanks, were incorporated into the final sequencing libraries.

In both conventional and HiMEx approaches, PCR products were pooled at approximately equimolar concentrations after quantification by Quant-iT PicoGreen dsDNA Assay Kit. The

pooled libraries underwent an additional purification step using 0.8× (vol:vol) magnetic beads before sequencing. Amplicon libraries were sequenced on Illumina MiSeq (PE300).

Testing HiMEx on standard microbial community

Beyond comparing marine microbial communities, we also evaluated potential extraction biases using a cellular mock community primarily composed of human microbiome bacteria with varying cellular abundances. Although all taxa were recovered using HiMEx (FTPIG), there was a noticeable bias against Gram-positive bacteria (Fig. S2) consistent with known biases from widely used extraction kits (https://www.zymoresearch.de/products/zymobiomics-microbial-community-standard). While this is an important consideration, the majority of marine microbial taxa are derived from Gram-negative lineages. Thus, given that Gram-positive bacteria may generally be more challenging to extract than marine bacteria (and yet were still recovered). These results, along with our results from the marine communities are encouraging. This test also helped to ensure low biomass detection by our HiMEx (FTPIG) extraction. The ZymoBIOMICS Spike-in Control I (High Microbial Load) was added to some samples before extraction in different proportions to estimate detection threshold of the HiMEx that were removed from the ASV table before visualization.

Amplicon bioinformatic analysis

Multiple extraction and PCR controls in addition to a mock community and spiked-in bacteria (for some samples) were included as internal controls and processed identically to environmental samples. This ensured comparability across sequencing runs while minimizing bias and contamination. After inspection, spike-in controls were removed from further analysis.

Demultiplexed amplicon sequences were trimmed with cutadapt v4.4 [7] implemented in QIIME2 v2024.10 [8], discarding any sequence pairs not containing the forward or reverse primer (error rate set to 0.2). Amplicon sequences were then split into 16S and 18S pools using bbsplit.sh [9] from the bbtools v4.4 (http://sourceforge.net/projects/bbmap/) against curated 16S/18S databases derived from SILVA 132 [10] and PR2 [11]. The 16S and 18S amplicons were then analysed in parallel to amplicon sequence variants (ASVs) using DADA2 [12] implemented in QIIME2. 16S ASVs were classified with qiime2 classify-vsearch plugin against the SILVA 138.1 database for chloroplast and mitochondria detection. Subsequently, the Greengenes2 database, version 2022.10, was used for classification for compatibility with the GTDB database and metagenomic analysis. ASVs identified as Mitochondria and unassigned reads were removed. Then, the ASV table was subdivided into prokaryotic 16S ASV table and chloroplast 16S ASV table (including all 16S ASVs identified as Chloroplast [13]). Chloroplast ASVs were further classified against PhytoRef database [14], and finally 19 ASVs identified as order "Embryophyceae", with 474 reads were removed from Chloroplast ASVs to only keep microalgae. 18S ASVs were assigned against the PR2 database. Decontamination analysis was first performed using “prevalence” method of Decontam R package v1.18.0 [15], via the function “isContaminant” which selects contaminant ASVs based on the prevalence (5%) of each sequence feature in true samples compared to the prevalence in negatives and controls (Tab. S13). Hence ASVs representing > 5% of each extraction blank were eliminated from the relevant extraction type. A final decision regarding the inclusion or exclusion of each contaminant ASV was made following a manual inspection and comparison with distributions in GBIF (www.gbif.org/occurrence/) to ensure accuracy based on where these taxa were commonly observed in marine waters and then considered non-contaminant. Ultimately, after decontamination, a total of 23 16S rRNA ASVs (16,721 reads) representing 1.6% of total

reads (15,80,941) were identified as potential contaminants (Tab. S13) and removed from the corresponding dataset. Chloroplast and 18S rRNA datasets did not show contamination under the same scrutiny. Subsequent to decontamination and removal of blank samples and samples below 1,000 for 16S rRNA and below and 100 reads for chloroplast and 18S rRNA, the total reads per sample varied between 1,000 to 83,675 reads/sample (n=84) for 16S rRNA, and between 100 and 28,502 for chloroplast (n=84) and 100 to 21,953 for 18S rRNA (n=53). A final number of 72, 76, and 53 samples remained in 16S rRNA, chloroplast and 18S rRNA datasets after rarefaction at 4,000, 100, and 100 sequence depth for prokaryotes, chloroplast and microbial eukaryotes downstream analysis, respectively. These thresholds were set after checking rarefaction curves, constructed using the rarecurve() function from vegan package and sample retention rates. Rarefaction curves visualize species richness as a function of sequencing depth enabling examination of the degree of sequence saturation. Although the rarefaction threshold of 100 for chloroplast 16S and 18S was not typically saturated, it was used to allow inclusion of a higher number of samples (Fig. S4). For the chloroplast 16S rRNA dataset an alternative rarefaction threshold of 500 read depth was also tested which showed similar results for PERMANOVA (Tab. S5-B1). Application of 500 read depth for 18S rRNA dataset was not feasible as it would result in the loss of either the conventional or HiMEx counterpart in most timepoints, rendering comparisons impossible (Tab. S5-C1).

Amplicon Statistical analysis

Assessing microbial community and diversity analysis

Bray-Curtis distances computed from the rarefied amplicon abundance tables were used to test for homogeneity of dispersion (beta dispersion), through vegan package [16] with 9,999 permutations

to determine the variability between replicates depending on extraction types or volumes. Generally variability between replicates increased in microvolumes and in FT and FTP extraction methods (Tab. S6). Then, microbial community variation at different timepoints was assessed using permutational analysis of variance (PERMANOVA) with obtained Bray-Curtis distance matrices. Since some timepoints were testing only three extraction methods, to have balanced designs, the dataset was analysed in three subsets with extraction method types equally distributed (Tab. S5.A-C). Surface seawater samples were significantly different at different timepoints. Temperature, salinity and time (month of sampling) and their interactions could explain 0.64, 0.57, and 0.43 of variation in the prokaryotic, chloroplast and microbial eukaryotes datasets, respectively (Tab. S5.A-C). To determine if statistical differences existed at the taxonomic levels between extraction methods and volumes, (PERMANOVA) was computed using the pairwise.adonis function on the subsets of rarefied amplicon abundance tables correcting for the impact of different timepoints as strata, and the *p* value was adjusted throughout (p.adjust = "Holm").

Although PERMANOVA did not show significant difference between conventional and microvolume extractions (Tab. S5.A-C), DESeq2 [17] was used to identify differentially abundant ASVs between conventional vs. FTPIG treatment of HiMEx in prokaryotes and conventional vs. FT, FTP and FTPIG in microalgae and microbial eukaryotes across all timepoints and within each timepoint. In the prokaryotic dataset higher enrichment of *Pelagibacter* was among the factors driving differences between HiMEx and conventional extraction (Tab. S9-A). *Pelagibacter*, the most dominant bacteria in the ocean, is classified as an ultramicrobacterium, with a cell volume of less than 0.1 μm^3 [18], which can potentially pass through 0.2-μm-pore-size filters [19]. In deeper oceanic areas (75 to 500 m) the *Pelagibacter*-like DNA has been reported to dominate the vesicle

fractions [20], complicating the interpretation of whether differential abundance reflects cellular DNA or extracellular DNA. However, this concern is likely minimal for surface seawater samples [20]. In addition, either lower number of particles due to higher stochasticity in microvolumes or differences in extraction efficiency of these methods on particle-attached bacteria could have caused higher enrichment of these bacteria in conventional extraction (Tab. S9-A).

Linear mixed models (LMM) from the R package lme4 [21] as described in Ghotbi *et al.* [22] were used to estimate the comparative and interactive effects of extraction methods and volumes on prokaryotes, microalgal and microbial eukaryotes diversity (Shannon indices). Timepoint (KFTno, Kiel Fjord Time series number, Tab. S8) was included as a fixed effect to account for the impact of environmental variables which were the main drivers of community clustering. In addition, either extraction method or volume was included also as a fixed effect to test for any additional methodological impact (Tab. S8). These parameters were also included as random effects to assess their contribution to the partitioning of variance. Pairwise comparisons between timepoints and extraction methods within each timepoint were conducted using estimated marginal means via the emmeans package, with Holm correction applied to adjust for multiple testing. The ggplot2 [23] and the DspikeIn R packages [24] were used for visualization of data. Statistical groupings for timepoints visualized using compact letter display (CLD) from the multcomp package to indicate significant differences between timepoints.

Microcosm experimental manipulation and sampling for metagenomic analysis

A pilot high-throughput microcosm experiment was conducted to examine how bloom demise (phytoplankton necromass) influences the composition and diversity of marine microbial communities across domains and viruses. Surface water from the Baltic Sea, including both whole

seawater and its free-living fraction, was kept as seawater control and also incubated with necromass derived from axenic cultures of *Chaetoceros calcitrans*. The *C. calcitrans* cell concentrate was prepared by gentle centrifugation of cells at the end of the exponential phase. After elution in fresh media cellular abundance was 8.29×10^6 cells/mL (via Gallios flow cytometry). Volumes of 800 µL were added to a deep well plate and subjected to freeze-thaw to obtain *C. calcitrans* necromass. Then 1200 µL of either 3-µm filtered or whole seawater was added to the necromass achieving a final concentration of 3.31×10^6 cells/mL. The deep well plate was incubated at 21 °C under a 14:10 hour light-dark cycle for eight days. Samples of 100 µL were collected daily from different treatments and control samples for metagenomic sequencing. For validation of HiMEx, samples collected from the microcosm experiment were compared with the initially collected 500 mL water samples (4 October) which underwent filter-based conventional extraction. Since cell abundances were not measured prior to experiment and extraction, counts were assumed to be 10^6 cells/mL in seawater samples and an order of magnitude higher in enriched treatments. Accordingly, ZymoBIOMICS Spike-in Control I were added to each sample equal to 200 in seawater samples ($\simeq$ 0.2% of assumed 10^6 cells/mL in seawater) and 20,000 ($\simeq$ 2% of assumed 10^7 cells/mL in enriched treatments). However, subsequent microscopy-based cell counts revealed higher actual abundances ($0.97 - 7.56 \times 10^6$) than initially estimated, which further reduced the proportion of spiked cells below the recommended threshold (1 to 10 %) and likely contributed to non-detection in seawater samples.

Sequencing for metagenome libraries

Sequencing libraries for metagenomes were prepared using one ng input with 12 to 15 amplification cycles for conventional vs. 10 µL lysate with 16 cycles for HiMEx, respectively. Briefly, DNA was tagmented in 50 µL reactions, amplified using the Phusion® Hot Start Flex DNA Polymerase (M0535, New England Biolabs) protocol, excluding DMSO according to the Hackflex protocol [25]. Forward and reverse primers included P5 or P7 adapters, 8-base-indexes, and partial overhangs matching to the transposome adapter TCGTCGGCAGCGTC or GTCTCGTGGGCTCGG, respectively. PCR began with an initial denaturation at 98 °C for 30 seconds, followed by 12 to 15 cycles of 10 seconds at 98 °C for conventional vs 16 cycles for HiMEx, 30 seconds at 62 °C, and 30 seconds at 72 °C, with a final extension at 72 °C for 5 minutes. After amplification, products underwent 0.6× size selection using AMPure XP magnetic beads, followed by bioanalyzer evaluation and an additional 0.6× bead clean-up to remove short DNA fragments. Blank samples were included in all PCR protocols and incorporated into the final sequencing libraries. Metagenome libraries were sequenced on an Illumina NovaSeq 6000 (2 x 150 bp paired reads).

Metagenome bioinformatic analysis

Raw sequences were quality-controlled using Trimmomatic (v0.39) [26] to remove adapter sequences, low-quality bases, and short reads. Specifically, reads with a Phred score below 30 were trimmed using a sliding window approach (50:30), and reads shorter than 50 bp were discarded. Post-trimming, read quality was assessed using FastQC [27] to ensure the removal of low-quality regions and adapter contamination. High-quality paired-end and unpaired reads were individually assembled using SPAdes (v3.11.1) [28], applying the following k-mer values: 21, 33,

55, 77, 99, and 127, with the option –sc. Assemblies were quality checked using MetaQUAST [29] (Fig. 2). Insert sizes of sequenced hackflex libraries were determined via the Spades assembler log files.

Contigs shorter than 5,000 bp were excluded from downstream analyses. Trimmed reads were mapped back to the assembled contigs using Bowtie2 (v2.3.4.3) [30]. To recover metagenome-assembled genomes (MAGs), three binning algorithms were utilized: MaxBin2 [31], MetaBAT2 [32], and CONCOCT [33]. The outputs from the three algorithms were consolidated using DASTool [34] to generate final high-quality bins for each sample. The quality of the resulting bins was assessed using CheckM (v1.1.2) [35] to estimate genome completeness and contamination. Bin quality was reported based on DASTool quality definitions (Tab. S12-C). Thus, bins were classified as high-quality draft (>=90% completeness) or draft (90% to 70% completeness) only if contamination levels were equal or below 5%. To remove redundant MAGs (i.e., to dereplicate MAGs) for all samples together, we used dRep (v2.6.0) with default setings [36] which identifies essentially identical genomes and selects the one of highest quality. A total number of 238 MAGs (high-, draft-, and low-quality) were recovered from all samples which were dereplicated into 93 MAGs (Tab. S12-C). All 238 MAGs were included in the analyses testing for significant differences in completeness and contig number between methods (Tab. S12. A-B). 138 of these MAGs were classified as high- or draft-quality based on DASTool criteria. Two MAGs identified as contamination were removed, resulting in a final number of 136 high- and draft-quality MAGs, which were subsequently dereplicated into 67 MAGs (Tab. S11-A).

For decontamination, blank samples were included in and incorporated into the final sequencing libraries. However, after metagenomic analysis workflow blank samples did not yield contigs of

at least 5kb (our lowest contig size threshold). Contigs (from the contaminant MAG classified as *Cutibacterium* obtained from amplicon analysis) found in the second timepoint of WSW-NCh and FSW-NCh, plus those two samples were removed from analysis (Fig. 2), though these contigs were typically undetected in the samples. All MAGs were classified taxonomically using GTDB-Tk (v2.3.2) [37] and the Genome Taxonomy Database (GTDB) release 214.1. The phylogenomic tree of the recovered MAGs was constructed using gtotree (v1.8.1) [38] with the IQtree (v2.1.11) [39], based on high quality draft and draft genomes. Anvi'o was utilized to calculate coverage data based on the Q2Q3 statistic. To enable this, a contigs database was generated within Anvi'o (v8) with anvi-gen-contigs-database, [40] followed by profiling of the samples via anvi-profile and subsequently merged with anvi-merge. Anvi'o Q2Q3 data was calculated via the anvi-summarize command. Q2Q3 data were normalized by the total number of base pairs in the paired-end reads using a per-gigabase scaling approach. This ensured that abundance values reflected microbial community composition across the samples, and these profiles were utilized in downstream statistical analyses to assess microbial community structure. In addition, an end-to-end detection and annotation of plasmids and viruses from contigs equal or larger than 5,000 bp was done through geNomad (v1.8.1) [41] using default settings. Viral sequence quality and completeness were assessed using CheckV [42] (database: checkv-db-v1.5) in end-to-end mode. After excluding proviruses, complete, high and medium quality viral contigs were selected for further analysis which resulted in 465 viral contigs (Tab. S14). To reduce sequence redundancy and dereplicate them, the viral sequences were clustered using cd-hit (4.8.1) [43] with a sequence identity threshold of 95% and an alignment coverage of 85%, with the longest contig being assigned as the representative sequence (VOTU, Tab. S14). 344 high and medium quality VOTUs remained for further analysis.

Enumeration of phytoplankton, prokaryotes, and viruses in the seawater samples

For field samples, at each timepoint, 10 mL of seawater was preserved with 0.25% glutaraldehyde incubated for 10–15 minutes at room temperature, and stored at -80 ºC. Upon thawing, 2 mL of sample was filtered onto 0.02 µm Anodisc filter (Whatman), and stained with 1:100 SYBR Green I following Patel *et al.* [44]. Anodiscs were mounted on to microscopy slides with 0.2% p-phenylenediamine anti-fade mounting medium and visualized on a Zeiss Axio Imager.Z2 Epifluorescence Microscope. Images were acquired in .czi format and analyzed using Fiji (v2.16.0) [45], a distribution of ImageJ bundled with essential plugins for biological imaging. Obtained images were imported via Bio-Format import option which preserved the embedded metadata, including scaling information (pixel size). Pixel size and image scale was verified using the Set Scale tool. For each sample, seven to 10 fields of view were selected at random across the filter depending on image quality. The area of each field of view was calculated using the Analyze > Measure function. Particle counts were performed using Analyze > Analyze Particles function, following manual signal to noise optimization. Particles were categorized into viral-, bacterial-, and phytoplankton-like groups based on relative size ranges, as defined by Patel *et al.* [39]. Phytoplankton identification was further validated using the chlorophyll fluorescence channel. Number of enumerated particles across six timepoints ranged from 5,669,647 to 50,362,628 for virus-like particles and from 974,372 to 7,558,153 for prokaryote-like particles and from 292 to 17,963 for phytoplankton-like particles (Tab. S1). Based on these data, the lowest bacterial and viral abundances were observed in early September, while the highest abundances occurred in mid-August. The lowest phytoplankton count was recorded in mid-November.

Supplementary References

1. Moisander PH, Beinart RA, Voss M, Zehr JP. Diversity and abundance of diazotrophic microorganisms in the South China Sea during intermonsoon. *ISME J* 2008; **2**: 954–967.
2. Demir-Hilton E, Sudek S, Cuvelier ML, Gentemann CL, Zehr JP, Worden AZ. Global distribution patterns of distinct clades of the photosynthetic picoeukaryote Ostreococcus. *ISME J* 2011; **5**: 1095–1107.
3. Bramucci AR, Focardi A, Rinke C, Hugenholtz P, Tyson GW, Seymour JR, et al. Microvolume DNA extraction methods for microscale amplicon and metagenomic studies. *ISME Communications* 2021; **1**: 1–5.
4. Parada AE, Needham DM, Fuhrman JA. Every base matters: assessing small subunit rRNA primers for marine microbiomes with mock communities, time-series and global field samples. *Environ Microbiol* 2016; **18**: 1403–1414.
5. McNichol J, Berube PM, Biller SJ, Fuhrman JA. Evaluating and Improving Small Subunit rRNA PCR Primer Coverage for Bacteria, Archaea, and Eukaryotes Using Metagenomes from Global Ocean Surveys. *mSystems* 2021; **6**: e0056521.
6. Yeh Y-C, McNichol JC, Needham DM, Fichot EB, Fuhrman JA. Comprehensive single-PCR 16S and 18S rRNA community analysis validated with mock communities and denoising algorithms. *bioRxiv* . 2019. , 866731
7. Martin M. Cutadapt removes adapter sequences from high-throughput sequencing reads. *EMBnet J* 2011; **17**: 10.
8. Bolyen E, Rideout JR, Dillon MR, Bokulich NA, Abnet CC, Al-Ghalith GA, et al. Reproducible, interactive, scalable and extensible microbiome data science using QIIME 2. *Nat Biotechnol* 2019; **37**: 852–857.

9. BBMap: A Fast, Accurate, Splice-Aware Aligner.
10. Yilmaz P, Parfrey LW, Yarza P, Gerken J, Pruesse E, Quast C, et al. The SILVA and 'All-species Living Tree Project (LTP)' taxonomic frameworks. *Nucleic Acids Res* 2013; **42**: D643–D648.
11. Guillou L, Bachar D, Audic S, Bass D, Berney C, Bittner L, et al. The Protist Ribosomal Reference database (PR2): a catalog of unicellular eukaryote small sub-unit rRNA sequences with curated taxonomy. *Nucleic Acids Res* 2013; **41**: D597–604.
12. Callahan BJ, McMurdie PJ, Rosen MJ, Han AW, Johnson AJA, Holmes SP. DADA2: High-resolution sample inference from Illumina amplicon data. *Nat Methods* 2016; **13**: 581–583.
13. Needham DM, Fuhrman JA. Pronounced daily succession of phytoplankton, archaea and bacteria following a spring bloom. *Nature microbiology* 2016; **1**: 16005.
14. Decelle J, Romac S, Stern RF, Bendif EM, Zingone A, Audic S, et al. PhytoREF: A reference database of the plastidial 16S rRNA gene of photosynthetic eukaryotes with curated taxonomy. *Mol Ecol Resour* 2015; **15**: 1435–1445.
15. Davis NM, Proctor DM, Holmes SP, Relman DA, Callahan BJ. Simple statistical identification and removal of contaminant sequences in marker-gene and metagenomics data. *Microbiome* 2018; **6**: 226.
16. R-project. org/package= vegan H, 2011. vegan: Community Ecology Package-R package version 1.17-8. *cir.nii.ac.jp* 2011.
17. Love MI, Huber W, Anders S. Moderated estimation of fold change and dispersion for RNA-seq data with DESeq2. *Genome Biol* 2014; **15**: 550.
18. Nakai R. Size matters: Ultra-small and filterable microorganisms in the environment. *Microbes Environ* 2020; **35**.

19. Lanclos VC, Rasmussen AN, Kojima CY, Cheng C, Henson MW, Faircloth BC, et al. Ecophysiology and genomics of the brackish water adapted SAR11 subclade IIIa. *ISME J* 2023; **17**: 620–629.

20. Linney MD, Eppley JM, Romano AE, Luo E, DeLong EF, Karl DM. Microbial sources of exocellular DNA in the ocean. *Appl Environ Microbiol* 2022; **88**: e0209321.

21. Bates D, Mächler M, Bolker B, Walker S. Fitting Linear Mixed-Effects Models using lme4. *arXiv [statCO]* . 2014.

22. Ghotbi M, Taghizadeh-Mehrjardi R, Knief C, Ghotbi M, Kent AD, Horwath WR. The patchiness of soil 13C versus the uniformity of 15N distribution with geomorphic position provides evidence of erosion and accelerated organic matter turnover. *Agric Ecosyst Environ* 2023; **356**: 108616.

23. Villanueva RAM, Chen ZJ. ggplot2: Elegant Graphics for Data Analysis (2nd ed.). *Measurement (Mahwah NJ)* 2019; **17**: 160–167.

24. Absolute abundance unveils Basidiobolus as a cross-domain bridge indirectly bolstering gut microbiome homeostasis.

25. Gaio D, Anantanawat K, To J, Liu M, Monahan L, Darling AE. Hackflex: Low-cost, high-throughput, Illumina Nextera Flex library construction. *Microbial Genomics* 2022; **8**.

26. Bolger AM, Lohse M, Usadel B. Trimmomatic: a flexible trimmer for Illumina sequence data. *Bioinformatics* 2014; **30**: 2114–2120.

27. Andrews S. FastQC: a quality control tool for high throughput sequence data. *(No Title)* 2010.

28. Bankevich A, Nurk S, Antipov D, Gurevich AA, Dvorkin M, Kulikov AS, et al. SPAdes: a new genome assembly algorithm and its applications to single-cell sequencing. *J Comput*

Biol 2012; **19**: 455–477.

29. Mikheenko A, Saveliev V, Gurevich A. MetaQUAST: evaluation of metagenome assemblies. *Bioinformatics* 2016; **32**: 1088–1090.
30. Langmead B, Salzberg SL. Fast gapped-read alignment with Bowtie 2. *Nat Methods* 2012; **9**: 357–359.
31. Wu Y-W, Simmons BA, Singer SW. MaxBin 2.0: an automated binning algorithm to recover genomes from multiple metagenomic datasets. *Bioinformatics* 2015; **32**: 605–607.
32. Kang DD, Li F, Kirton E, Thomas A, Egan R, An H, et al. MetaBAT 2: an adaptive binning algorithm for robust and efficient genome reconstruction from metagenome assemblies. *PeerJ* 2019; **7**: e7359.
33. Alneberg J, Bjarnason BS, de Bruijn I, Schirmer M, Quick J, Ijaz UZ, et al. Binning metagenomic contigs by coverage and composition. *Nat Methods* 2014; **11**: 1144–1146.
34. Sieber CMK, Probst AJ, Sharrar A, Thomas BC, Hess M, Tringe SG, et al. Recovery of genomes from metagenomes via a dereplication, aggregation and scoring strategy. *Nature Microbiology* 2018; **3**: 836–843.
35. Parks DH, Imelfort M, Skennerton CT, Hugenholtz P, Tyson GW. CheckM: assessing the quality of microbial genomes recovered from isolates, single cells, and metagenomes. *Genome Res* 2015; **25**: 1043–1055.
36. Olm MR, Brown CT, Brooks B, Banfield JF. dRep: a tool for fast and accurate genomic comparisons that enables improved genome recovery from metagenomes through de-replication. *ISME J* 2017; **11**: 2864–2868.
37. Chaumeil P-A, Mussig AJ, Hugenholtz P, Parks DH. GTDB-Tk v2: memory friendly classification with the Genome Taxonomy Database. *bioRxiv* . 2022.

38. Lee MD. GToTree: a user-friendly workflow for phylogenomics. *Bioinformatics* 2019; **35**: 4162–4164.

39. Minh BQ, Schmidt HA, Chernomor O, Schrempf D, Woodhams MD, von Haeseler A, et al. IQ-TREE 2: New models and efficient methods for phylogenetic inference in the genomic era. *Mol Biol Evol* 2020; **37**: 1530–1534.

40. Eren AM, Esen ÖC, Quince C, Vineis JH, Sogin ML, Delmont TO. Anvi'o: An advanced analysis and visualization platform for 'omics data. *PeerJ* . 2015.

41. Camargo AP, Roux S, Schulz F, Babinski M, Xu Y, Hu B, et al. Identification of mobile genetic elements with geNomad. *Nat Biotechnol* 2024; **42**: 1303–1312.

42. Nayfach S, Camargo AP, Schulz F, Eloe-Fadrosh E, Roux S, Kyrpides NC. CheckV assesses the quality and completeness of metagenome-assembled viral genomes. *Nat Biotechnol* 2021; **39**: 578–585.

43. Fu L, Niu B, Zhu Z, Wu S, Li W. CD-HIT: accelerated for clustering the next-generation sequencing data. *Bioinformatics* 2012; **28**: 3150–3152.

44. Patel A, Noble RT, Steele JA, Schwalbach MS, Hewson I, Fuhrman JA. Virus and prokaryote enumeration from planktonic aquatic environments by epifluorescence microscopy with SYBR Green I. *Nat Protoc* 2007; **2**: 269–276.

45. Schindelin J, Arganda-Carreras I, Frise E, Kaynig V, Longair M, Pietzsch T, et al. Fiji: an open-source platform for biological-image analysis. *Nat Methods* 2012; **9**: 676–682.

46. Zweifel UL, Hagstrom A. Total counts of marine bacteria include a large fraction of non-nucleoid-containing bacteria (ghosts). *Appl Environ Microbiol* 1995; **61**: 2180–2185.

Supplementary Tables

Tab. S1. Values represent averages from 7 to 10 counted fields for each sample.

Tab. S2. Amplicon analysis Metadata file.

Tab. S3. ASV table (16S rRNA gene) of standard microbial community with different cell number input. Some samples were spiked with specific cell numbers of internal control which were removed before analysis and making the plot.

Tab.S4. Statistical output of Kruskal-Wallis test on sequence depth.

Tab. S5-A1. PERMANOVA to compare variability in prokaryotic microbial community over different timepoints of KFT (Kiel Fjord time series) after rarefaction at 4000 sequence depth (72 samples).

Tab. S5.A2. PERMANOVA to compare variability in prokaryotic microbial community among different extraction methods after rarefaction at 100 sequence depth (72 samples).

Tab. S5.A3. PERMANOVA to compare variability in prokaryotic microbial community among different extracted volumes (72 samples).

Tab. S5-B1. PERMANOVA to compare variability in microalgae community over different timepoints of KFT (Kiel Fjord time series) after rarefaction at 100 sequence depth (76 samples).

Tab. S5.B2. PERMANOVA to compare variability in microalgae community among different extraction methods (76 samples).

Tab. S5.B3. PERMANOVA to compare variability in microalgae community among different extracted volumes (76 samples).

Tab. S5-C1- PERMANOVA to compare variability in microbial eukaryotes community over different timepoints of KFT (Kiel Fjord time series) after rarefaction at 100 sequence depth (59 samples).

Tab. S5.C2. PERMANOVA to compare variability in microbial eukaryotes community among different extraction methods after rarefaction at 100 sequence depth (59 samples).

Tab. S5.C3. PERMANOVA to compare variability in microbial eukaryotes community among different extracted volumes after rarefaction at 100 sequence depth (59 samples).

Tab. S6 Permutation test for homogeneity of multivariate dispersions in microbial communities via Tukey multiple comparisons of means 95% family-wise confidence level.

Tab. S7. Primer construction for the single-step rRNA gene amplicon amplifications.

Tab. S8-A. Diversity analysis (α-diversity_Shannon indices) of prokaryotic community.

Tab. S8-B. Diversity analysis (α-diversity_shannon indices) of microalgae community.

Tab. S8-C. Diversity analysis (α-diversity_Shannon indices) of microbial eukaryotes.

Tab. S8-D. Diversity analysis (α-diversity_Shannon indices) of prokaryotic community for different extraction volumes.

Tab. S8-E. Diversity analysis (α-diversity_shannon indices) of microalgae community for different extraction volumes

Tab. S8-F. Diversity analysis (α-diversity_Shannon indices) of microbial eukaryotes for different extraction volumes.

Tab. S9-A. Differential abundance of prokaryotic taxa at the ASV level between "conventional" and "FTPIG" extraction methods. "conventional" is used as the reference condition. Only those with p.adj <0.05 are considered significant.

Tab. S9-B1. Differential abundance of microalgae taxa at the ASV level between "conventional" and "FTPIG" extraction methods. "conventional" is used as the reference condition. Only those with p.adj <0.05 are considered significant.

Tab. S9-B2. Differential abundance of microalgae taxa at the ASV level for timepoint KFTno=85 (12 Aug) between "conventional" and "FTP" extraction methods. "conventional" is used as the reference condition. Only those with p.adj <0.05 are considered significant.

Tab. S9-B3. Differential abundance of microalgae taxa at the ASV level for timepoint KFTno=85 (12 Aug) between "conventional" and "FT" extraction methods. "conventional" is used as the reference condition. Only those with p.adj <0.05 are considered significant.

Tab. S9-C1. Differential abundance of microbial eukaryotes at the ASV level between "conventional" and "FTPIG" extraction methods. "conventional" is used as the reference condition. Only those with p.adj <0.05 are considered significant.

Tab. S9-C2. Differential abundance of microbial eukaryotes at the ASV level between "conventional" and "FT" extraction methods. "conventional" is used as the reference condition. Only those with p.adj <0.05 are considered significant.

Tab. S10-A. Cost breakdown of extraction work flows (€/sample).

Tab. S10-B. Time required for extraction of 96 samples. The preprocessing step for the conventional method includes filtration of 500 mL marine water samples using a 12-place manifold under moderate vacuum followed by rinsing of the filtration apparatus, whereas for the HiMEx workflows, preprocessing involves only sample transfer into a 96 well plate.

Tab. S11-A. Number of high- and draft- quality MAGs vs dereplicated high- amd draft- quality MAGs obtained from conventional vs HiMEx method. A total of 138 high and draft quality MAGs were recovered from all samples. Two MAGs identified as contamination were removed, resulting in a final set of 136 MAGs.

Tab. S11-B. Number of complete, high- and medium- quality viral contigs vs dereplicated ones obtained from conventional vs HiMEx method.

Tab. S12-A. Comparison of completeness of all 238 MAGs (high, draft and low quality) obtained from conventional vs HiMEx method. Benjamini–Hochberg (BH) method used for p-value adjustment.

Tab. S12-B. Comparison of contig number of all 238 MAGs (high, draft and low quality) obtained from conventional vs HiMEx method. Benjamini–Hochberg (BH) method used for p-value adjustment.

Tab. S12-C. Number and quality of all MAGs (238) obtained from conventional vs HiMEx method. MAGS quality reported according to DASTool evaluation.

Tab. S13. Contaminants list based on 5% prevalence of 16S dataset in blank samples after manual control. The last 10 ASVs belong to the Zymo standard community, to ensure removal of any contamination from positive controls.

Tab. S14. Number and quality of viral contigs and VOTUs obtained from conventional vs HiMEx method.

Tab. S15. Ratios of coverage-based viral to bacterial abundance (VBR) in conventional vs HiMEx of high-throughput experiment.

Supplementary Figures

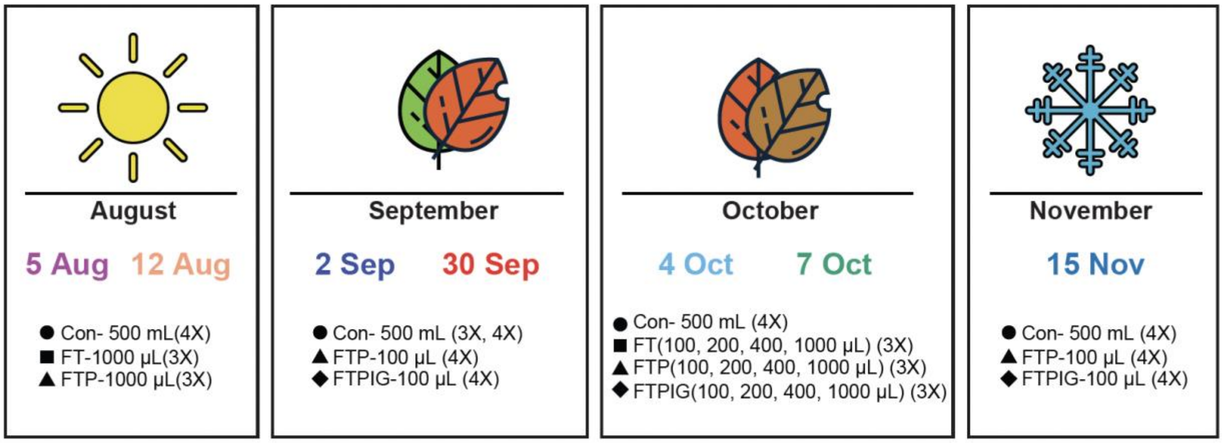

Fig. S1. An incomplete factorial design across four months and seven timepoints was applied, where balanced subsets of HiMEx method (FT, FTP, FTPIG) and volume (100, 200, 400, 1000 µL) combinations were compared with conventional extraction (500 mL) as a control in each timepoint.

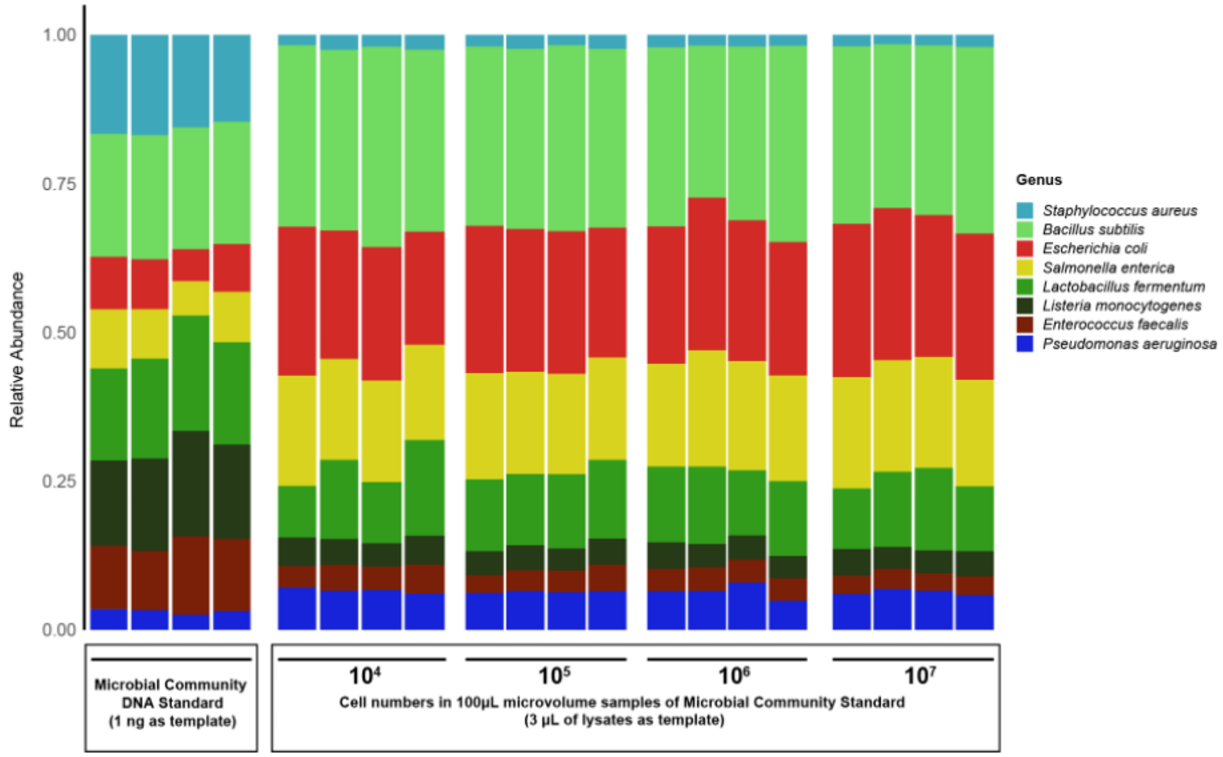

Fig. S2. Barplots showing 16S rRNA gene amplicon analysis of HiMEx extractions tested on a gradient of low to medium biomass of standard microbial community (ZymoBIOMICS, D6300), in tandem with DNA standard (ZymoBIOMICS, D6305) (Tab. S13). The cell concentrations selected aimed to assess the sensitivity and ability of HiMEx to amplify cells at lower and higher concentration than typically reported in seawater [46] and our samples (~10^6). We acknowledge a bias against some Gram positive bacteria, which should be considered while applying this method in environments where these bacteria are more abundant like soil.

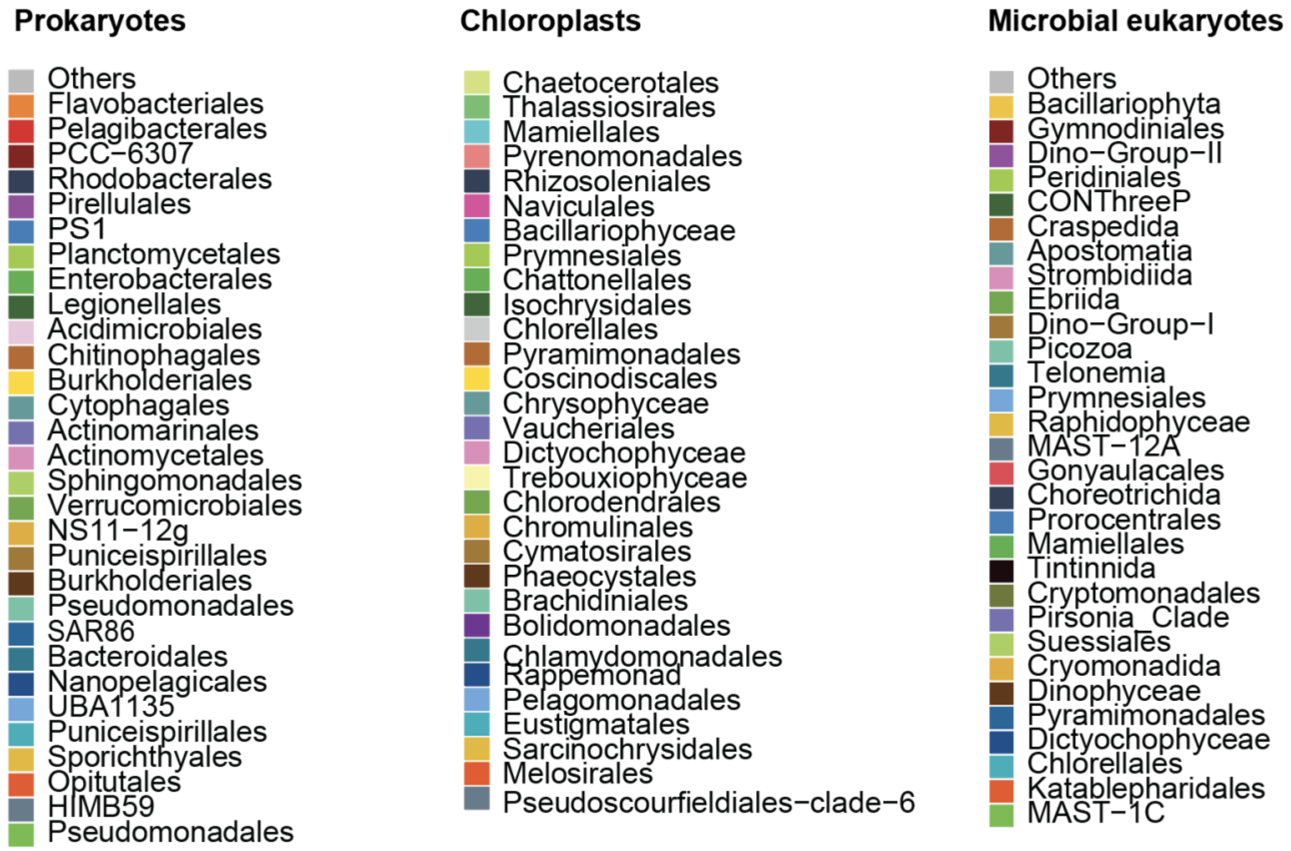

Fig. S3. Color coding for the top 30 orders of microbial community composition in prokaryotes, microalgae, and microbial eukaryotes presented in Fig. 1-B.

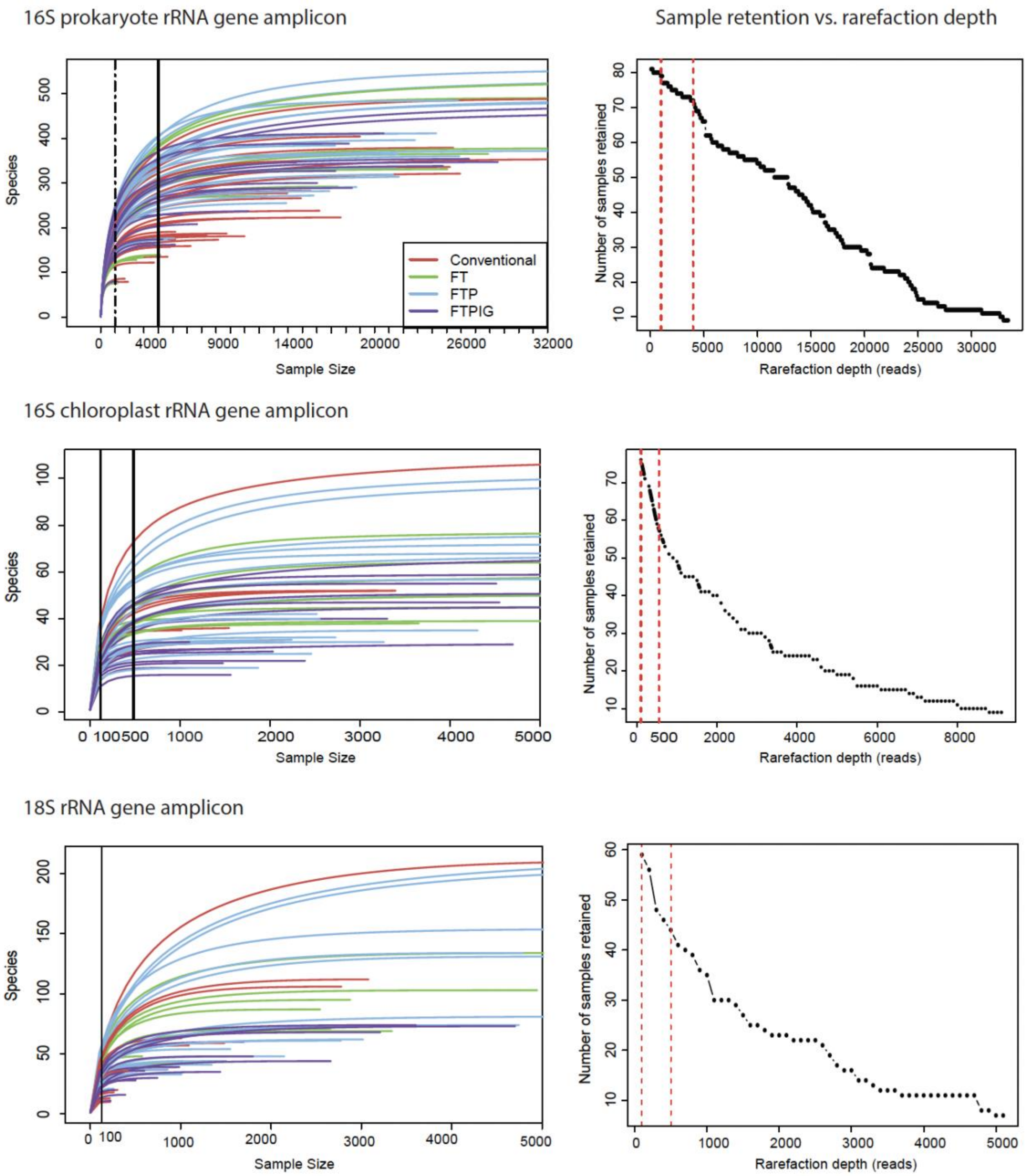

Fig. S4. Rarefaction curves and sample retention rate of 16S rRNA, chloroplast, and 18S rRNA gene sequencing data. The solid black lines indicate the rarefaction depths used for downstream analysis. A dashed black line is shown when a different depth was applied solely for visualization

purposes. In the right-hand plots the dashed red line indicates the number of samples retained at each rarefaction depth. For the chloroplast 16S rRNA dataset an alternative rarefaction threshold of 500 read depth was also tested which showed similar results for PERMANOVA (Tab. S5. B1).

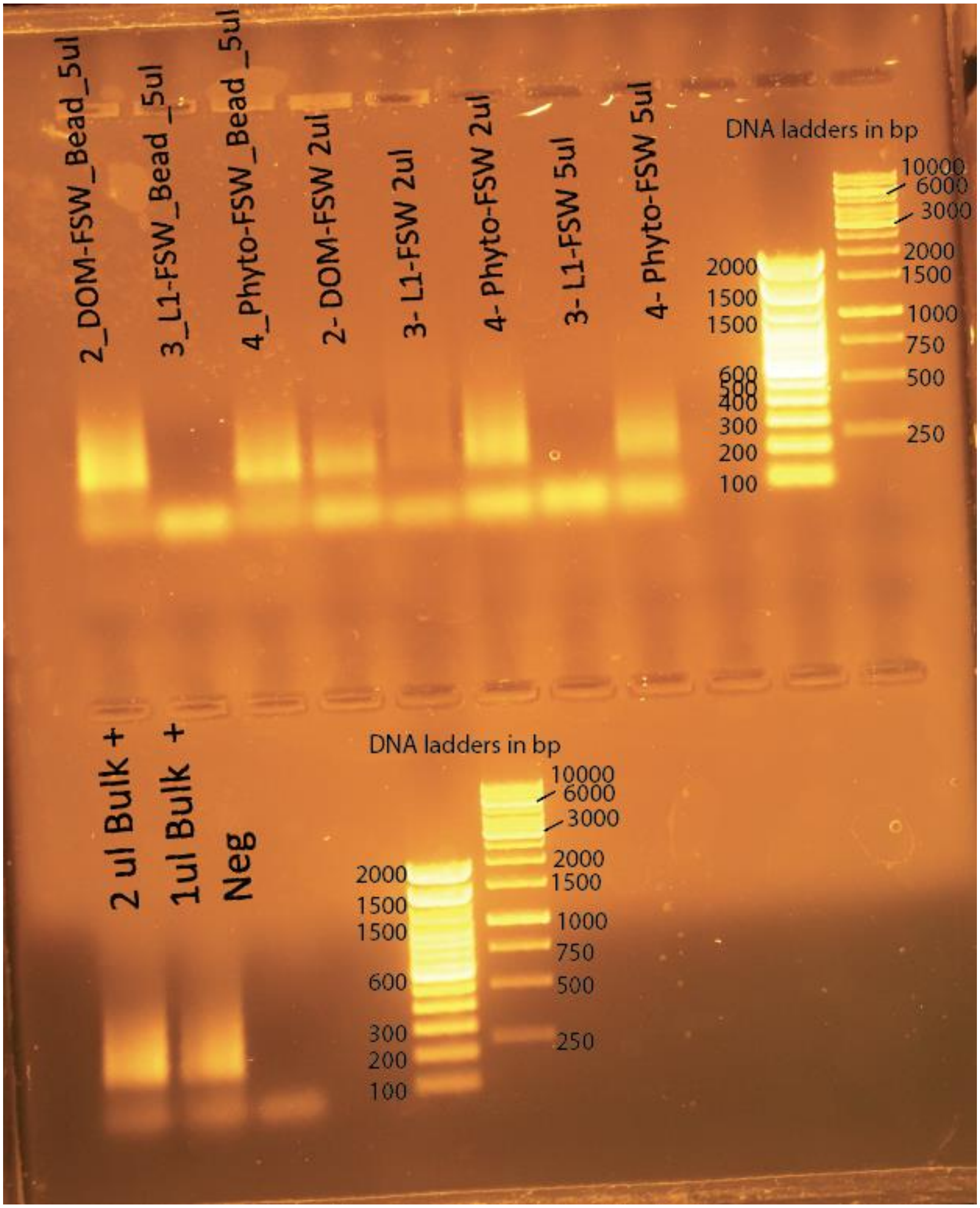

Fig. S5. Demonstration of positive amplification via the Hackflex metagenome protocol across variable input quantities and purifications. The gel image shows test samples after HiMEx extraction and following amplification via the Hackflex protocol (16 cycles of PCR) using

different volumes of lysate (2 to 5 μL) as template for direct PCR and 10 μL using AMPure XP magnetic beads purification (concentrated as 5 μL). According to the original Hackflex paper, the average insert size of libraries prepared using Hackflex with size selection as done here is 416 to 433bp, which is obtained after size-selection with beads and sequencing. In our case, after sequencing, our insert sizes were determined to range from 373 to 503 bp for HiMEx extractions and 363-442 bp for conventional extraction, which is within a reasonable range compared to the original Hackflex paper. The gel indicates that the peak of the fragment size was ~250 bp before size selection, and thus the subsequent size selection by design shifted the sequenced insert size to be longer. DOM_FSW: Free living fraction of seawater incubated with DOM of *Chaetoceros calcitrans* cultures. L1_FSW: Free living fraction of seawater incubated with L1(phytoplankton medium). Phyto_FSW: Free living fraction of seawater incubated with *Chaetoceros calcitrans* necromass. Bead: Sample purified using Ampure beads. Bulk+: Conventional extractions positive control. Neg: Negative control

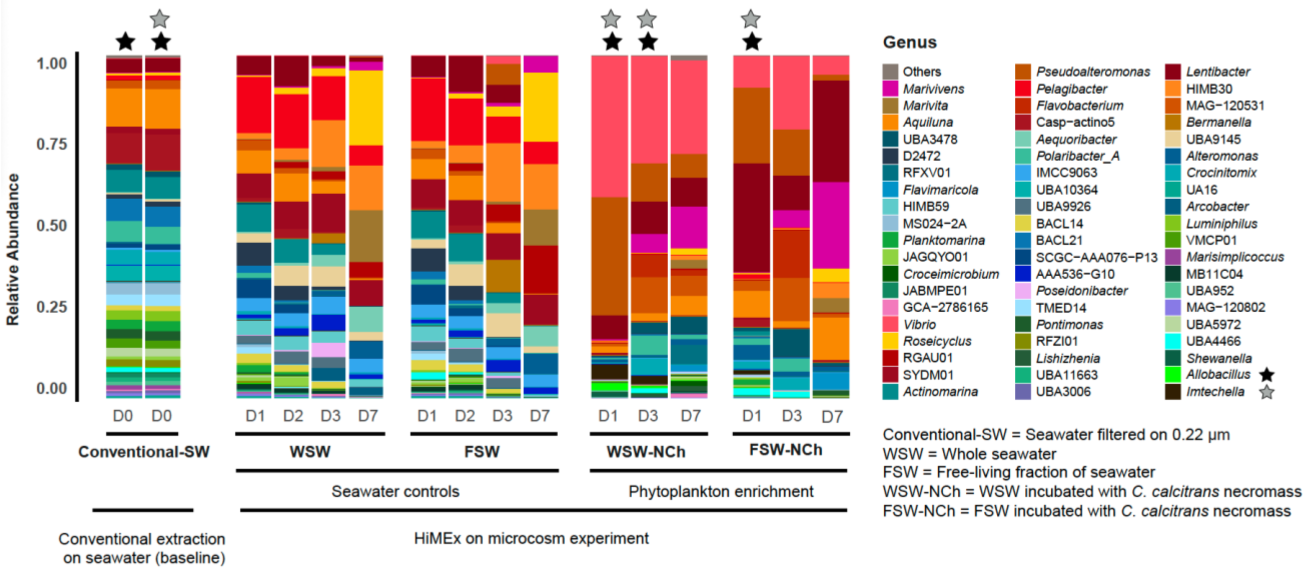

Fig. S6. Taxonomic diversity recovered by conventional and HiMEx via shotgun metagenomics (Hackflex). Conventional metagenomes were sequenced from the Day 0 (baseline) samples, and HiMEx metagenomes were sequenced after incubation with phytoplankton necromass (*Chaetoceros calcitrans*). Incubation of the natural bacterial community with phytoplankton necromass clearly increased the abundance of *Vibrio*, *Pseudoalteromonas, Marivivens, and Lentibacter* especially in whole seawater samples containing particle-associated bacteria. Asterisks above the bars indicate samples where the two spiked bacterial taxa, *Allobacillus halotolerans* and *Imtechella halotolerans*, were detected above a relative abundance of > 0.001.

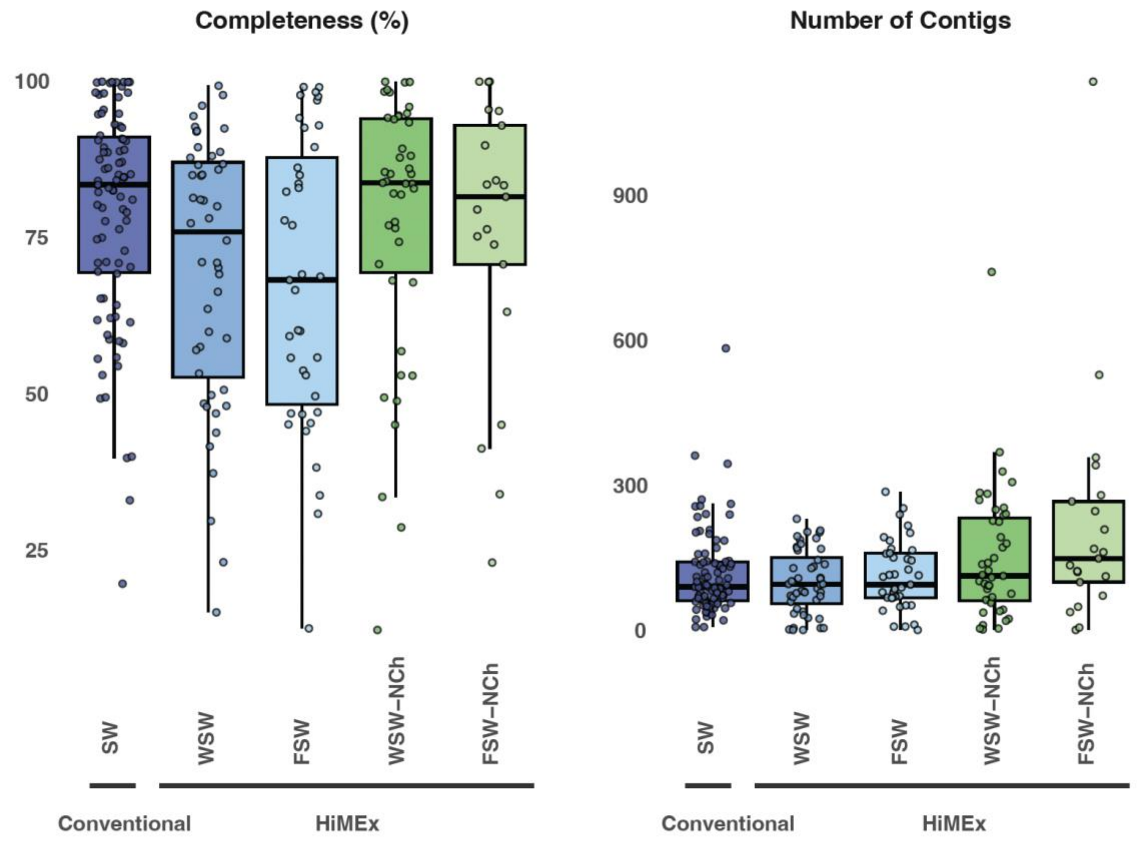

Fig. S7. Boxplots showing completeness (%) and number of contigs per MAGs for conventional extraction on seawater vs. HiMEx extraction on seawater and microcosm experiment. MAG completeness differed slightly across conventional extraction and HiMEx (Kruskal–Wallis $H(4) = 10.5$, $p = 0.033$) (Tab. S12-A), but post-hoc pairwise comparisons using Dunn's test with Benjamini–Hochberg correction revealed no statistically significant differences (all $p.adj > 0.05$). The number of contigs per MAG did not significantly differ among methods (Kruskal–Wallis $H(4) = 7.57$, $p = 0.109$) (Tab. S12-B).
